# Emerging concepts and recent advances in renal cell neoplasia

**DOI:** 10.1007/s00428-026-04695-9

**Published:** 2026-09-05

**Authors:** Kiril Trpkov, Abbas Agaimy

**Affiliations:** 1 Diagnostic and Molecular Pathology, Cumming School of Medicine, University of Calgary, Calgary, AB Canada; 2 Institute of Pathology, Erlangen University Hospital, Friedrich Alexander University of Erlangen-Nuremberg, Erlangen, Germany; 3 Comprehensive Cancer Center, European Metropolitan Area Erlangen-Nuremberg (CCC ER-EMN), Institute of Pathology, Erlangen University Hospital, Friedrich Alexander University of Erlangen-Nuremberg, Erlangen, Germany

Renal tumors exhibit remarkable biological complexity and represent a challenging diagnostic area in pathology that had undergone significant changes in the last two decades, reflected in the dynamic evolution of their taxonomy, presented in the recent WHO classifications. In this issue of Virchows Archiv, we feature original studies, reviews and brief report studies that highlight some of the latest developments on several renal tumor entities. Recent evidence, including the study by Zhao et al. in the current issue of Virchows Archiv (https://doi.org/10.1007/s00428-025-04362-5) indicates that renal hemangioblastoma (RHB) is a distinct morphologic pattern within the spectrum of renal cell carcinoma with fibromyomatous stroma (RCC-FMS). Several recent publications examined the relationship between RHB and RCC-FMS, mostly in a sporadic setting, but also in a setting of Tuberous Sclerosis Complex (TSC), to elucidate and highlight the similarities in their pathology, immunohistochemistry, and molecular findings. In sum, the evidence supports the concept of a continuum of one entity with indolent biological behavior. This concept suggests that HB-like areas, including pure RHB, represent a variant within a TSC/mTOR pathway–altered RCC-FMS family, and are different from the classical clear cell RCC and from the central nervous system HB. In this issue, we also present a critical review of the literature by Baranova and Trpkov (https://doi.org/10.1007/s00428-026-04510-5) on RHB and RCC-FMS-HB that further supports the notion that RHB and HB-like areas in RCC-FMS should be considered components of the spectrum of RCC-FMS rather than separate entities, based on their shared clinical, histological, immunophenotypic, and molecular features. Thus, the journey of this entity that started in 1993 in the publication by Canzonieri et al. [1] who initially described RCC FMS as “mixed renal tumor with carcinomatous and fibroleiomyomatous components” and was subsequently labelled in the literature under various names, will ultimately have a successful conclusion, accompanied by elimination of RHB as a distinct entity. We hope that these new insights will result in its recognition as a new entity in the next WHO classification of renal tumors.

Another entity that recently attracted attention is the FLCN-mutated tumor, typically seen in Birt-Hogg-Dubé (BHD) syndrome, which is an autosomal dominant disorder caused by germline inactivation of the folliculin gene (FLCN). In a multi-institutional study by Siegmund et al (https://doi.org/10.1007/s00428-026-04588-x), one of the largest molecularly confirmed cohorts of FLCN-mutated tumors, they conducted an integrated histologic, immunohistochemical, and targeted next-generation sequencing analyses. They found that *FLCN* biallelic inactivation was uniformly found in all patients with tumors that typically exhibited ‘conventional’ histology consisting of solid or nested growth with a characteristic mosaic population of eosinophilic and clear cells, low-grade nuclear features, and indolent behavior. Interestingly, they also found two tumors with ‘non-conventional’ histology including tubulocystic and tubulopapillary architecture that had advanced stage at presentation, including one with metastatic disease. Similarly, in another study in this issue, Wang et al (https://doi.org/10.1007/s00428-026-04693-x) studied six FLCN-mutated high-grade RCC with ‘non-conventional’ features, exhibiting papillary/tubulopapillary and solid growth patterns. Three of the six patients developed distant metastases, two of which died of disease. Their findings expand the morphologic spectrum of FLCN-mutated renal tumors and further document the existence of an aggressive subset of such tumors. These findings support vigilant FLCN testing in morphologically overlapping high-grade papillary tumors that are difficult to classify based on morphology and immunohistochemistry. Along with other recently presented evidence, their findings support the recognition of FLCN-driven tumors as a distinct, molecularly defined entity withing the spectrum of eosinophilic/oncocytic renal tumors.

TSC/mTOR pathway alterations have been documented in several recently recognized renal tumor types, such as eosinophilic vacuolated tumor (EVT), low-grade oncocytic tumor of the kidney (LOT), eosinophilic solid and cystic (ESC) RCC and RCC with fibromyomatous stroma. Importantly, most renal tumors with TSC/mTOR pathway mutations have been reported to be indolent, with only rare tumors exhibiting metastatic potential. Kwon et al. describes two cases of MTOR-mutated eosinophilic RCCs with loss of chromosome 1 that showed metastatic spread (https://doi.org/10.1007/s00428-025-04363-4). Their findings add to the literature on MTOR-mutated eosinophilic RCCs with loss of chromosome 1 that have distinct morphologic, immunohistochemical, and molecular features, as well as metastatic potential, and further expand the spectrum of TSC/mTOR pathway-mutated renal tumors.

Papillary renal cell neoplasm with reverse polarity (PRNRP) is another recently recognized renal tumor characterized by papillary architecture lined by a single layer of low-grade eosinophilic cells with apically located nuclei. However, PRNRP has been considered as a morphological pattern of papillary RCC in the latest WHO classification. Marletta et al. (https://doi.org/10.1007/s00428-026-04536-9) performed a comparative analysis of 15 PRNRPs and 16 eosinophilic oncocytic papillary RCCs (E/OPRCC) evaluating their histopathological, immunophenotypic, interphase cytogenetic, and molecular profiles. They found that PRNRP exhibited distinct morphologic features, consistent GATA3 positivity, uniform *KRAS* mutations and lacked trisomies of chromosomes 7 and 17, and uniform indolent behavior. Their findings further support the classification of PRNRP as a distinct renal entity, separated from both classic papillary RCC and its eosinophilic/oncocytic morphological variant.

Glycoprotein nonmetastatic melanoma protein B (GPNMB) is a transmembrane protein that has recently emerged as a valuable diagnostic marker for various renal neoplasms exhibiting FLCN/TSC/mTOR-TFE3/TFEB alterations. The sensitivity and specificity of GPNMB immunostain are in some cases suboptimal and the interpretation in ambiguous cases may be difficult. Li et al. (https://doi.org/10.1007/s00428-026-04646-4) found that GPR143, a transmembrane protein regulated by MiT transcription factors, was highly expressed in a subset of RCCs with FLCN/TSC/mTOR-TFE3/TFEB alterations. Of note, despite concordant GPR143 and GPNMB immunoreactivity in most renal neoplasms with FLCN/TSC/mTOR-TFE3/TFEB alterations, diffuse GPR143 immunostain was observed in some cases with negative or focal GPNMB labeling, suggesting that GPR143 could be used as a surrogate marker for such diagnostically challenging tumors.

Succinate dehydrogenase (SDH)-deficient RCC and fumarate hydratase (FH)-deficient RCC are rare but clinically significant renal entities bearing important implications not only on the affected patients but also for their families. Fanelli et al. (https://doi.org/10.1007/s00428-026-04431-3) conducted a nationwide, web-based survey among the members of the Italian Study Group of Uropathology (GIUP) to evaluate the real-world awareness, diagnostic pathways, and the test availability across Italian centers regarding these two entities. They found that recognition of these entities in Italy is primarily morphology-driven but was constrained by uneven access to confirmatory IHC, particularly 2SC, and to molecular assays. Their findings argue for introduction of harmonized diagnostic algorithms, regional reference laboratory networks, and routine involvement of molecular tumor boards, supported by targeted educational efforts, to standardize the practice and to improve patient pathways from morphologic suspicion to genetic counselling and tailored surveillance.

Finally, this issue of Virchows Archiv contains several articles that feature a variety of uncommon epithelial, mesenchymal and hematolymphoid renal neoplasms, presenting in-depth studies on their molecular pathogenesis and highlighting diagnostic pitfalls. We also present brief reports on rare and emerging yet not well-delineated molecular subtypes of RCC. We hope that not only GU pathologists, but also the broader readership of the journal will enjoy this special issue and will find the articles presented herein of educational and scientific value, both for routine diagnostic work and for inspiring future research activities on renal neoplasia.

## Data Availability

N/A.
